# Programmable Gene Knockdown in Diverse Bacteria Using Mobile‐CRISPRi

**DOI:** 10.1002/cpmc.130

**Published:** 2020-12-17

**Authors:** Amy B. Banta, Ryan D. Ward, Jennifer S. Tran, Emily E. Bacon, Jason M. Peters

**Affiliations:** ^1^ Pharmaceutical Sciences Division, School of Pharmacy University of Wisconsin‐Madison Madison Wisconsin; ^2^ Great Lakes Bioenergy Research Center, Wisconsin Energy Institute University of Wisconsin‐Madison Madison Wisconsin; ^3^ Laboratory of Genetics University of Wisconsin‐Madison Madison Wisconsin; ^4^ Department of Bacteriology University of Wisconsin‐Madison Madison Wisconsin; ^5^ Department of Medical Microbiology and Immunology University of Wisconsin‐Madison Madison Wisconsin

**Keywords:** *Bacillus subtilis*, biofuels, conjugation, CRISPR‐Cas9, CRISPRi, *Escherichia coli*, ESKAPE pathogens, functional genomics, *Listeria monocytogenes*, *Zymomonas mobilis*

## Abstract

Facile bacterial genome sequencing has unlocked a veritable treasure trove of novel genes awaiting functional exploration. To make the most of this opportunity requires powerful genetic tools that can target all genes in diverse bacteria. CRISPR interference (CRISPRi) is a programmable gene‐knockdown tool that uses an RNA‐protein complex comprised of a single guide RNA (sgRNA) and a catalytically inactive Cas9 nuclease (dCas9) to sterically block transcription of target genes. We previously developed a suite of modular CRISPRi systems that transfer by conjugation and integrate into the genomes of diverse bacteria, which we call Mobile‐CRISPRi. Here, we provide detailed protocols for the modification and transfer of Mobile‐CRISPRi vectors for the purpose of knocking down target genes in bacteria of interest. We further discuss strategies for optimizing Mobile‐CRISPRi knockdown, transfer, and integration. We cover the following basic protocols: sgRNA design, cloning new sgRNA spacers into Mobile‐CRISPRi vectors, Tn*7* transfer of Mobile‐CRISPRi to Gram‐negative bacteria, and ICE*Bs1* transfer of Mobile‐CRISPRi to Bacillales. © 2020 The Authors.

**Basic Protocol 1**: sgRNA design

**Basic Protocol 2**: Cloning of new sgRNA spacers into Mobile‐CRISPRi vectors

**Basic Protocol 3**: Tn*7* transfer of Mobile‐CRISPRi to Gram‐negative bacteria

**Basic Protocol 4**: ICE*Bs1* transfer of Mobile‐CRISPRi to Bacillales

**Support Protocol 1**: Quantification of CRISPRi repression using fluorescent reporters

**Support Protocol 2**: Testing for gene essentiality using CRISPRi spot assays on plates

**Support Protocol 3**: Transformation of *E. coli* by electroporation

**Support Protocol 4**: Transformation of CaCl_2_‐competent *E. coli*

## INTRODUCTION

**Figure 1:**
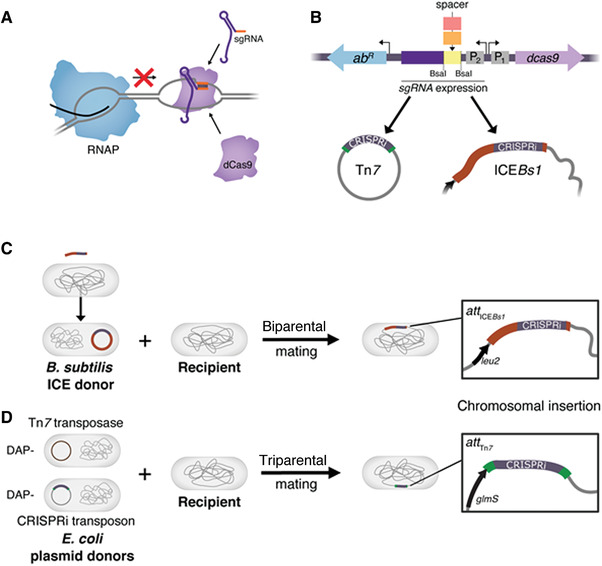
CRISPRi and Mobile‐CRISPRi. (**A**) CRISPRi represses transcription by sterically blocking RNA polymerase (RNAP) elongation. (**B**) Mobile‐CRISPRi is a modular system containing sgRNAs and *dcas9* that is inserted into a Tn*7* vector or the ICE*Bs1* element for transfer to recipient bacteria. Spacers targeting new genes can be cloned into BsaI sites upstream of the sgRNA. (**C**) ICE*Bs1* Mobile‐CRISPRi transfers to recipient bacteria via biparental mating and integrates into the genome downstream of the *leu2* tRNA gene. (**D**) Tn*7* Mobile‐CRISPRi transfers to recipient bacteria via triparental mating with donors containing either a plasmid with Tn*7* transposon ends flanking the CRISPRi components or a plasmid expressing Tn*7* transposase genes. Tn*7* integrates into the recipient genome downstream of the *glmS* gene.

CRISPRi (Fig. 1A) is a contemporary gene‐perturbation strategy with substantial advantages over classic genetic approaches (e.g., gene deletions, transposon mutagenesis). First, CRISPRi is programmable (Qi et al., 2013): to specify a gene for knockdown, one need only alter the first 20 nucleotides (nt) of the sgRNA (known as the “spacer”) to match the target gene (provided that there is a nearby protospacer‐adjacent motif, or PAM; see Strategic Planning). This programmability makes it straightforward to construct single‐gene knockdowns or knockdown libraries targeting defined sets of genes (Liu et al., 2017; Peters et al., 2016) or all genes in the genome (Lee et al., 2019; Rousset et al., 2018; Wang et al., 2018; Yao et al., 2020). Because the sgRNA spacer uniquely identifies the target gene, spacers can act as barcodes to obtain counts of individual knockdown strains and measure their fitness in pooled growth experiments. Second, CRISPRi is inducible and titratable, and therefore can be used to target essential genes (Li et al., 2016; Peters et al., 2016). The timing and extent of CRISPRi knockdown can be controlled by expressing the *sgRNA* and *dcas9* genes from inducible promoters. Further, knockdown gradients can be achieved by expressing CRISPRi components from constitutive promoters of differing strengths (Qu et al., 2019), by using truncated spacers (Vigouroux, Oldewurtel, Cui, Bikard, & Teeffelen, 2018), or by systematically mutating the sgRNA to introduce mismatches between the spacer and target gene that reduce knockdown efficacy to a predictable extent (Hawkins et al., 2020). Finally, CRISPRi can be multiplexed to target several genes in the same cell by cloning arrays of sgRNAs with different spacers (Ellis, Kim, & Machner, 2020; Peters et al., 2016; Reis et al., 2019). These advantages suggest that CRISPRi will become a common tool for bacterial functional genomics in the near future.

Despite the utility of CRISPRi, its widespread adoption in bacteria has been limited by a lack of generalizable systems. To facilitate the use of CRISPRi in diverse bacteria, we developed Mobile‐CRISPRi—a suite of modular vectors that transfer by conjugation and stably integrate into the genomes of recipient bacteria (Peters et al., 2019). All Mobile‐CRISPRi vectors contain (1) *dcas9*, (2) an sgRNA with either a targeting spacer or restriction sites for cloning new spacers, and (3) an antibiotic resistance marker for selection in recipient bacteria (Fig. 1B). The modularity of Mobile‐CRISPRi enables facile cloning of sgRNA libraries. There are two general sets of Mobile‐CRISPRi vectors: ICE*Bs1*‐based vectors for Gram‐positive bacteria related to *Bacillus subtilis* (i.e., order Bacillales) that integrate downstream of the *leu2* tRNA (Fig. 1C), and Tn*7*‐based vectors for Gram‐negative bacteria that integrate downstream of the *glmS* gene (Fig. 1D). Tn*7* Mobile‐CRISPRi vectors transfer from a diaminopimelic acid (DAP)‐dependent donor strain of *Escherichia coli* to recipient strains of interest via the RP4 conjugation machinery and require a helper plasmid for integration (also transferred from *E. coli*) that expresses the Tn*7* transposition genes (*tnsABCD*; triparental mating). ICE*Bs1* Mobile‐CRISPRi vectors are first integrated into the genome copy of ICE*Bs1* in a *B. subtilis* donor strain using natural competence transformation and are then transferred to recipient strains of interest through induction of the ICE*Bs1* conjugation machinery (biparental mating). Integration of Mobile‐CRISPRi downstream of *glmS* or *leu2* does not impart a fitness defect in the strains tested (although Tn*7* insertion might not be neutral in all species, and expression of *dcas9* may be toxic in some bacteria; see Commentary).

Mobile‐CRISPRi transfer, integration, and knockdown efficiencies vary by recipient strain for reasons that are sometimes but not always understood (Banta, Enright, Siletti, & Peters, 2020; Peters et al., 2019; Qu et al., 2019). To rapidly assess Mobile‐CRISPRi knockdown efficacy, we developed “test” vectors for both the Tn*7* and ICE*Bs1* systems that contain either an *mRFP* or an *sfGFP* gene (encoding monomeric red fluorescent protein or superfolder green fluorescent protein, respectively) and an sgRNA targeting *mRFP* or *sfGFP* that allows knockdown to be easily measured in live cells using a fluorometer or flow cytometer. The modular nature of Mobile‐CRISPRi enables straightforward knockdown optimization approaches, such as replacing the sgRNA and *dcas9* promoters or *dcas9* ribosome‐binding site with native regulatory sequences from the recipient bacterium (see Commentary). Thus far, we have demonstrated Mobile‐CRISPRi transfer/integration and knockdown in several Gram‐negative (γ‐Proteobacteria: *E. coli*, *Enterobacter cloacae*, *Enterobacter aerogenes*, *Pseudomonas aeruginosa*, *Klebsiella pneumoniae*, *Vibrio casei*, *Acinetobacter baumannii*, *Salmonella enterica*, and *Proteus mirabilis* [Peters et al., 2019]; α‐Proteobacteria: *Zymomonas mobilis* [Banta et al., 2020]) and Gram‐positive species (Firmicutes: *B. subtilis*, *Listeria monocytogenes*, *Staphylococcus aureus*, and *Enterococcus faecalis* [Peters et al., 2019]).

Here, we describe the following basic protocols: (1) sgRNA design for selecting and computationally optimizing guide spacers (Basic Protocol 1), (2) cloning of new sgRNA spacers into Mobile‐CRISPRi vectors to target specific genes for knockdown (Basic Protocol 2), (3) Tn*7* transfer of Mobile‐CRISPRi to Gram‐negative bacteria via conjugation with *E. coli* donors (Basic Protocol 3), and (4) ICE*Bs1* transfer of Mobile‐CRISPRi to Bacillales via conjugation with a *B. subtilis* donor (Basic Protocol 4). We also provide the following support protocols: (1) quantification of CRISPRi repression using fluorescent reporters that measure knockdown in live cells (Support Protocol 1), (2) testing for gene essentiality using CRISPRi spot assays on plates to examine the effects of CRISPRi gene knockdown on plating efficiency (Support Protocol 2), (3) transformation of *E. coli* by electroporation (Support Protocol 3), and (4) transformation of CaCl_2_‐competent *E. coli* to facilitate cloning sgRNA spacers and create donor strains for Mobile‐CRISPRi mating (Support Protocol 4).

#### Biosafety caution

*CAUTION*: Follow all biosafety requirements relevant to the microorganism under investigation. See Burnett et al. (2009) for more information.

## STRATEGIC PLANNING

Before beginning CRISPRi experiments in a new strain, one must first design sgRNA spacers against target genes (see Basic Protocol 1). In general, desirable sgRNAs are on target (have only one binding site in the genome) and have high efficacy (although lower‐efficacy guides may be useful for targeting essential genes; Hawkins et al., 2020; Vigouroux et al., 2018). To design on‐target guides, we use a strategy developed by the Weissman and Gross labs that takes into account the DNA‐binding preferences of the dCas9‐sgRNA complex to score guide specificity (Gilbert et al., 2014; Peters et al., 2019). For instance, noncomplementarity between the sgRNA and a target site proximal to the PAM (the sgRNA “seed” region) has a strong negative effect on binding, whereas PAM‐distal mismatches (e.g., 15‐20 nt away from the PAM) have less impact on binding and may result in off‐target effects. To identify and eliminate off‐target guides, we strongly recommend designing sgRNA spacers for the entire genome at once (see Basic Protocol 1). If no closed genome sequence exists for the strain of interest, it is not possible to predict off‐target sgRNA activity; therefore, gene‐knockdown phenotypes must be confirmed by multiple guides. Because bacterial genomes are relatively small, most sgRNA spacers have high specificity (i.e., a maximum specificity score of 39 in our code below). sgRNA knockdown efficacy is complex and the subject of ongoing research (Calvo‐Villamañán et al., 2020), although some general rules have emerged. The clearest rule is that guides targeting the nontemplate strand of the gene (antisense, or “anti” in our code below) have much higher efficacy than template‐targeting guides (Bikard et al., 2013; Qi et al., 2013); the mechanistic underpinnings of this effect are unknown. Early observations suggested that sgRNAs targeting the 5′ ends of genes are more efficacious, but subsequent studies have shown that guide efficacy is constant across genes on average (Cui et al., 2018; Rousset et al., 2018). Nonetheless, we tend to prefer spacers that target toward the 5′ ends of genes because the effects of the CRISPRi transcription block on translation are unclear. Targeting promoters with CRISPRi can also be effective, but we prefer to target genes because of the lack of promoter location data in the majority of bacteria and the fact that spacer distribution is more limited in intergenic regions (i.e., there are fewer NGG PAM sequences in AT‐rich promoter regions).

## Basic Protocol 1 sgRNA DESIGN

This protocol describes how to design an exhaustive list of 20‐nucleotide sgRNA spacers using a command‐line interpreter. The process is streamlined and should be modified by changing the environment variable “ACC_NO” to specify a chromosomal accession number. Steps 1 and 2 are only required in the first setup and should be omitted afterwards.

### Materials

Linux‐ or Unix‐compatible computer with Internet access and the following software:
  Conda (https://docs.conda.io/projects/conda/en/latest/)
  GitHub (https://github.com/)
  Excel or other spreadsheet management software

### Download dependencies for sgRNA design script

1 Prepare a conda environment, “sgrna_design”, to host the sgRNA design scripts. It is only necessary to perform environment creation once.
conda create ‐n sgrna_design ‐c bioconda `bowtie=1.2.3' biopython pysam entrez‐direct git `python>3'
2 Retrieve the “sgrna_design” project from GitHub, then move into the newly created directory. It is only necessary to perform project download once.
git clone https://github.com/ryandward/sgrna_design.git && cd sgrna_design

### Activate Conda environment

3 Activate the environment to load script dependencies This is required every time a new terminal window is opened.
conda activate sgrna_design

### Obtain genome sequence data from NCBI using the accession number

4 If not known, consult the NCBI Nucleotide Database (see Internet Resources) to locate the accession number corresponding to the chromosome of interest, and adjust the environment variable “ACC_NO” accordingly. This example uses the *E. coli* MG1655 chromosome “U00096.3”; this number will serve as the template for the names of all other files.
ACC_NO="U00096.3"
5 Retrieve and save the GenBank chromosome file (.gb file)—here automatically named U00096.3.gb. This command also issues a warning if NCBI returns an empty response and may be run multiple times as needed.
efetch ‐db nuccore ‐format gb ‐id $ACC_NO > ${ACC_NO}.gb && file ${ACC_NO}.gb | grep ‐iq ascii && echo "File contains data, continue." || echo "Empty file, retry this step."

### Run sgRNA design script

6 Run the main python script, producing a tab‐separated variable file corresponding to the accession number followed by "_sgrna". Upon successful completion, this command also confirms the name of the results file—here "U00096.3_sgrna.tsv".
./build_sgrna_library.py ‐‐input_genbank_genome_name ${ACC_NO}.gb ‐‐tsv_output_file ${ACC_NO}_sgrna.tsv && echo "Output saved as ${ACC_NO}_sgrna.tsv"
  These results can be accessed by navigating to the present working directory and viewed using a spreadsheet utility, text editor, or command‐line interpreter tool (e.g., cat).
  Additional documentation is available at the GitHub repository sgrna_design (see Internet Resources at end of article).

## Basic Protocol 2 CLONING NEW sgRNA SPACERS INTO MOBILE‐CRISPRi VECTORS

**Figure 2:**
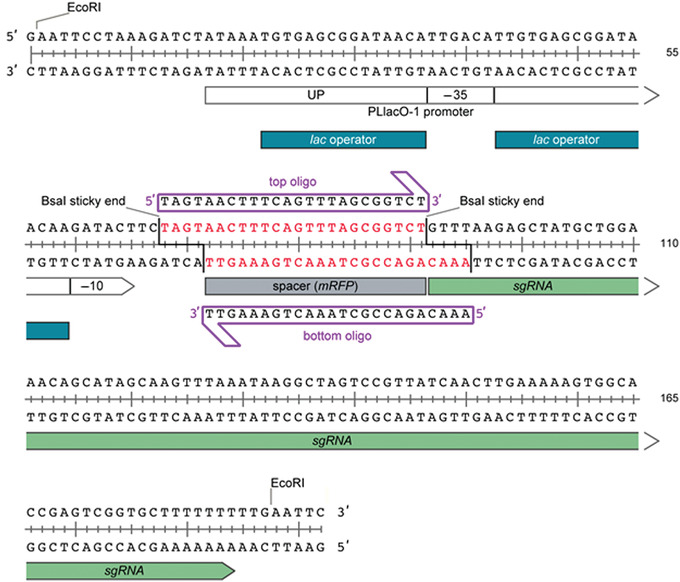
sgRNA spacer cloning. Shown here is the sgRNA module from the Mobile‐CRISPRi plasmid pJMP1339. Annealed oligos with BsaI‐compatible sticky ends are ligated into the BsaI‐cut vector (BsaI recognition sites are lost in the cloning process). This figure depicts a spacer targeting *mRFP*, but 20‐nt spacer sequences targeting any gene of interest can be cloned using this protocol.

This protocol describes the construction of Mobile‐CRISPRi vectors encoding an sgRNA spacer targeting a gene of interest. Two oligonucleotides are designed such that when annealed, they form the desired sgRNA spacer sequence with overhangs enabling ligation into a BsaI‐digested Mobile‐CRISPRi plasmid (Fig. 2). The resulting plasmid, which contains the entire Mobile‐CRISPRi system encoding the sgRNA, dCas9, and antibiotic resistance marker between Tn*7* transposon ends, can be used as a donor for transposition of the system into the recipient *att*_Tn_ *_7_* site.

### Materials

Mobile‐CRISPRi plasmid DNA (see Table 1)
BsaI‐HF‐v2 restriction enzyme and 10× CutSmart buffer (NEB R3733)
DNA spin‐purification kit (e.g., NEB T1030 or Zymo Research D4003)
Custom oligonucleotides (Top and Bottom, each 100 µM; see Basic Protocol 1, Strategic Planning, and Fig. 2)
T4 DNA ligase with 10× buffer (e.g., NEB M0202)
1 mM ATP (e.g., Thermo R0441, diluted 100×)
100 mM dithiothreitol (DTT; see recipe)
Electrocompetent *E. coli* cells (*pir* ^+^ strain, BW25141; Support Protocol 3)
LB agar with 100 µg/ml ampicillin agar plates
Liquid LB medium with 100 µg/ml ampicillin
Plasmid DNA miniprep kit (e.g., Thermo GeneJet kit K0502)

**Table 1:** Mobile‐CRISPRi Plasmids

JMP no.	Addgene no.	Rep.	Antibiotic res.*a*	sgRNA prom.	sgRNA	dCas9 prom.	dCas9	Comment
**ICE*Bs1* system**								
pJMP1333	119268	*mRFP*	amp^R^, kan^R^	Pveg	*mRFP* (NT1)	Pxyl/tet	Spy::3Xmyc	mRFP “test” (– sgRNA)
pJMP1335	119269	*mRFP*	amp^R^, kan^R^	None	None	Pxyl/tet	Spy::3Xmyc	mRFP “test” (+ sgRNA)
pJMP1337	119270	None	amp^R^, kan^R^	Pveg	None (BsaI)	Pxyl/tet	Spy::3Xmyc	For cloning new sgRNAs
**Tn*7* system**								
pJMP1039	119239	NA	amp^R^, N/A	N/A	N/A	N/A	N/A	Expresses Tn*7* transposase
pJMP1183	119254	*mRFP*	amp^R^, kan^R^	PLlacO1	*mRFP* (NT1)	PLlacO1	Spy::3Xmyc	mRFP “test” (+ sgRNA)
pJMP1185	119255	*mRFP*	amp^R^, kan^R^	None	None	PLlacO1	Spy::3Xmyc	mRFP “test” (– sgRNA)
pJMP1187	119256	*mRFP*	amp^R^, kan^R^	PLlacO1	*mRFP* (NT1)	PLlacO1	HSA Spy::3Xmyc	mRFP “test” (+ sgRNA)
pJMP1189	119257	*mRFP*	amp^R^, kan^R^	None	None	PLlacO1	HSA Spy::3Xmyc	mRFP “test” (– sgRNA)
pJMP1339	119271	None	amp^R^, kan^R^	PLlacO1	None (BsaI)	PLlacO1	HSA Spy::3Xmyc	For cloning new sgRNAs
pJMP1354	119275	None	amp^R^, tmp^R^	PLlacO1	None (BsaI)	PLlacO1	HSA Spy::3Xmyc	For cloning new sgRNAs
pJMP1356	119276	None	amp^R^, cm^R^	PLlacO1	None (BsaI)	PLlacO1	HSA Spy::3Xmyc	For cloning new sgRNAs
pJMP1358	119277	None	amp^R^, spc^R^	PLlacO1	None (BsaI)	PLlacO1	HSA Spy::3Xmyc	For cloning new sgRNAs
pJMP1360	119278	None	amp^R^, gent^R^	PLlacO1	None (BsaI)	PLlacO1	HSA Spy::3Xmyc	For cloning new sgRNAs
pJMP2754	160666	*sfGFP*	amp^R^, gent^R^	PLlacO1	None (BsaI)	PLlacO1	Spy::3Xmyc	sfGFP “test” (– sgRNA)
pJMP2774	160667	*sfGFP*	amp^R^, gent^R^	PLlacO1	Gmc6 (*sfgfp*)	PLlacO1	Spy::3Xmyc	sfGFP “test” (+ sgRNA)
pJMP2782	160668	None	amp^R^, gent^R^	PLlacO1	None (BsaI)	PLlacO1	Spy::3Xmyc	For cloning new sgRNAs
pJMP2820	160669	*sfGFP*	amp^R^, cm^R^	PLlacO1	None (BsaI)	PLlacO1	Spy::3Xmyc	sfGFP “test” (– sgRNA)
pJMP2822	160670	*sfGFP*	amp^R^, kan^R^	PLlacO1	None (BsaI)	PLlacO1	Spy::3Xmyc	sfGFP “test” (– sgRNA)
pJMP2824	160671	*sfGFP*	amp^R^, spc^R^	PLlacO1	None (BsaI)	PLlacO1	Spy::3Xmyc	sfGFP “test” (– sgRNA)
pJMP2832	160672	*sfGFP*	amp^R^, cm^R^	PLlacO1	Gmc6 (*sfgfp*)	PLlacO1	Spy::3Xmyc	sfGFP “test” (+ sgRNA)
pJMP2834	160673	*sfGFP*	amp^R^, kan^R^	PLlacO1	Gmc6 (*sfgfp*)	PLlacO1	Spy::3Xmyc	sfGFP “test” (+ sgRNA)
pJMP2836	160674	*sfGFP*	amp^R^, spc^R^	PLlacO1	Gmc6 (*sfgfp*)	PLlacO1	Spy::3Xmyc	sfGFP “test” (+ sgRNA)
pJMP2844	160675	None	amp^R^, cm^R^	PLlacO1	None (BsaI)	PLlacO1	Spy::3Xmyc	For cloning new sgRNAs
pJMP2846	160676	None	amp^R^, kan^R^	PLlacO1	None (BsaI)	PLlacO1	Spy::3Xmyc	For cloning new sgRNAs
pJMP2849	160677	None	amp^R^, spc^R^	PLlacO1	None (BsaI)	PLlacO1	Spy::3Xmyc	For cloning new sgRNAs

a Resistance(s) of recipient *E. coli*. amp^R^, ampicillin resistance; cm^R^, chloramphenicol resistance; gent^R^, gentamycin resistance; kan^R^, kanamycin resistance; spc^R^, spectinomycin resistance; tmp^R^, trimethoprim resistance.

Rep., reporter; prom., promoter. All plasmids have the R6K ori (replicate only in *pir* ^+^ strains); gmc6 encodes *sfGFP* sgRNA (CATCTAATTCAACAAGAATT); *mRFP* (NT1) encodes *mRFP* sgRNA (AACTTTCAGTTTAGCGGTCT); HSA Spy::3Xmyc dCas9 is human codon optimized, “None (BsaI)” sgRNA has a BsaI cloning site for insertion of new sgRNA encoding DNA; PLlacO1 promoters are regulated by *lacIq*; and Pxyl/tet promoter is regulated by TetR.

### Prepare BsaI‐digested Mobile‐CRISPRi plasmid

1 Digest 1 µg plasmid DNA by setting up the following reaction mixture:

**Table:** 

Component	Volume
10× CutSmart buffer (NEB)	5 µl
Plasmid DNA (100 ng/µl)	10 µl
BsaI‐HF‐v2 (NEB)	1 µl
Deionized H_2_O	34 µl
Total	50 µl.
  Adjust DNA and water volume as necessary according to DNA concentration. Scale reaction as necessary to ensure that plasmid DNA concentration does not exceed 25% of reaction and according to the total amount of digested plasmid needed for planned ligations (∼50 ng digested plasmid/ligation).
2 Incubate 2‐8 hr at 37°C to digest.
3 Incubate 20 min at 80°C to heat inactivate.
4 Purify the digested plasmid using a DNA spin‐purification kit according to the manufacturer's protocol. Elute in 10 µl.
  Use a kit that allows elution in low volumes. Heating the elution buffer to 50°C may increase yield.

### Anneal oligonucleotides

5 Set up each oligonucleotide annealing reaction:

**Table:** 

Component	Volume
10× CutSmart Buffer (NEB)	5 µl
Oligonucleotide‐Top (100 µM)	1 µl
Oligonucleotide‐Bottom (100 µM)	1 µl
dH_2_O	43 µl
Total	50 µl.
6 Incubate 5 min at 95°C to denature DNA.
7 Cool ∼15 min to room temperature to anneal oligonucleotides.
8 Dilute 1:40 in deionized water.

### Ligate BsaI‐digested plasmid and annealed oligonucleotides

9 Set up each ligation:

**Table:** 

Component	Volume
10× T4 DNA ligase buffer	1 µl
1 mM ATP	1 µl
100 mM DTT	1 µl
T4 DNA ligase	0.5 µl
1:40 diluted annealed oligonucleotides (step 8)	2 µl
50 ng BsaI‐digested plasmid (step 4)	*X* µl
Deionized water	*X* µl
Total	10 µl.
  Adjust volumes of water and plasmid as needed depending on concentration of plasmid so that the total reaction volume equals 10 µl. For increased efficiency and accuracy, set up a premix for multiple reactions that includes all components except annealed oligonucleotides, and then transfer aliquots of premix into to tubes and add annealed oligonucleotides.
  Always include a plasmid vector only/no annealed oligonucleotide ligation as a negative control to ensure complete digestion of plasmid DNA. A positive control reaction can be set up if desired using the mRFP targeting oligos shown in Figure 2.
10 Incubate 1‐2 hr at room temperature to ligate.
11 Incubate 15 min at 65°C to heat inactivate.

### Transformation

12 Follow Support Protocol 3 to transform 1 µl of ligation mix into *E. coli* BW25141 and select on LB‐ampicillin agar plates.
  Compare the transformants from the ligations that had annealed oligos to the digested plasmid only negative control. The negative control should have few or no colonies (<10% the amount for the other ligations). If there is a high background on the negative control plate, digest the plasmid again and repeat the ligation and transformation.

### Extract plasmid DNA and confirm sequence

13 Pick an isolated colony into 5 ml LB + ampicillin medium in a culture tube and incubate cultures ∼16 hr at 37°C, shaking at 250 rpm or on a roller drum.
14 Pellet cells from entire 5‐ml culture and extract plasmid DNA using a kit following the manufacturer's instructions.
  The Mobile‐CRISPRi plasmids are fairly large (∼10‐12 kb) and low copy. Extracting the entire 5‐ml culture and eluting in ∼40 µl typically yields ∼100 ng/µl plasmid DNA. Warming the elution buffer to 50°C and waiting ∼2 min after applying the buffer to the column before spinning may increase yield.
15 Sequence the region of the plasmid near the insertion site to confirm the insertion and the fidelity of the cloning.

## Basic Protocol 3 Tn*7* TRANSFER OF MOBILE‐CRISPRi TO GRAM‐NEGATIVE BACTERIA

This protocol transfers the Tn*7*‐based Mobile‐CRISPRi system into the chromosome of a Gram‐negative bacterium of interest. *E. coli* donor strains have a chromosomal copy of the RP4 transfer machinery to mobilize the Tn*7* plasmids. A plasmid with a Tn*7* transposon carrying CRISPRi components and a second plasmid encoding Tn*7* transposition genes are transferred to recipient bacteria by triparental mating. In the recipient, transposition proteins integrate the CRISPRi system into the recipient genome downstream of the *glmS* gene. Selection on plates lacking DAP eliminates the DAP‐dependent *E. coli* donors, whereas R6K ori plasmids are lost because they cannot replicate in recipient cells that lack the *pir* gene. Antibiotic selection results in retention of only the recipients with an integrated CRISPRi system. Once integrated into the chromosome, the Mobile‐CRISPRi system is stable without further antibiotic selection in all organisms tested so far. This method has been used with a variety of recipient γ‐Proteobacteria, including *Acinetobacter*, *Enterobacter*, *Escherichia*, *Klebsiella*, *Proteus*, *Pseudomonas*, *Salmonella*, *Shewanella*, and *Vibrio*, as well as the α‐Proteobacterium *Zymomonas*, and is likely to be adaptable to a wider range of bacteria.

### Materials

Plasmid DNA: Mobile‐CRISPRi‐encoding plasmids (Tn*7* version; Table 2)
LB medium, with additions of diaminopimelic acid (DAP) and/or ampicillin, as appropriate
LB agar plates, with appropriate additions, prewarmed to 37°C
Culture medium and agar plates suitable for growth of the recipient bacterium (with addition of antibiotics, as appropriate, depending on Mobile‐CRISPRi‐encoded resistance)
1× phosphate‐buffered saline (PBS; e.g., Fisher BP399500, diluted 1:10)

Cellulose filters (e.g., MF‐Millipore HAWG01300)
Tweezers, sterilized in ethanol

**Table 2:** Strains for Mobile‐CRISPRi (Tn*7* version)

Strain	JMP no.	Genotype	Selection	Comment	Ref.
*E. coli* BW25141	sJMP146	∆(*araD‐araB*)567, ∆*lacZ4787*(::*rrnB‐3*), ∆(*phoB‐phoR*)580, λ‐, *gal*U95, *∆uidA3::pir* ^+^, *recA1*, *endA9*(Δins)::FRT, rph‐1, ∆(*rhaD*‐*rhaB*)568, *hsdR514*	LB	*E. coli pir* ^+^ “cloning strain”	Datsenko & Wanner (2000)
*E.coli* WM6026	sJMP424	*lacIq*, *rrnB3*, DEl*acZ4787*, *hsdR514*, DE(*araBAD*)567, DE(*rhaBAD*)568, *rph‐1 att‐lambda::pAE12‐del* (*oriR6K/cat*::frt5), *Δ4229*(*dapA*)::frt(DAP^–^), Δ(*endA*)::frt, *uidA*(Δ*MluI*)::*pir*(wt), *attHK::pJK1006::Δ1/2*(ΔoriR6K‐*cat*::frt5, Δ*trfA*::frt)	LB + DAP	*E. coli pir* ^+^ “mating strain”	Blodgett et al. (2007)

### Prepare Mobile‐CRISPRi donor strains for Tn7 transposition

1 To make *E. coli* donor strains, transfer Mobile‐CRISPRi‐encoding plasmids (Tn*7* version) to *E. coli* mating strain (WM6026) following Support Protocol 3 or 4. Select on LB + 300 µM DAP + 100 µg/ml ampicillin.
  All Mobile‐CRISPRi plasmids have amp^R^ encoded on their backbone, so ampicillin can be used for selection during culture of the plasmid‐containing strain. When selecting for Tn7 transposition in your recipient (step 10 below), select instead for the transposon‐encoded antibiotic resistance.
  Transformation by electroporation (Support Protocol 3) is faster for a small number of plasmids, but transformation of CaCl_2_‐competent cells (Support Protocol 4) may be more suitable for high‐throughput strain construction.
2 Prepare stock strains in medium + 15% glycerol and store at −80°C.

### Tn7 transposition of Mobile‐CRISPRi

3 Streak strains onto agar plates from −80°C stocks to obtain isolated colonies. For *E. coli* donor strains, use LB + 300 µM DAP + 100 µg/ml ampicillin agar plates and incubate ∼14‐18 hr at 37°C. For recipient strain, use appropriate medium and culture conditions.
  Obtain biosafety approval before transferring Mobile‐CRISPRi to plant or animal pathogens, and perform all steps with appropriate biological containment for recipient strains.
4 Inoculate liquid cultures from a single colony and grow to saturation. For *E. coli* donor strains, use 5 ml LB + 300 µM DAP + 100 µg/ml ampicillin medium in a culture tube and incubate ∼14‐18 hr at 37°C with aeration (shaking at 250 rpm or on drum roller). For recipient strain, use appropriate medium, culture conditions, and culture volume to obtain between 1‐ and 10‐fold the number of recipient cells compared to the *E. coli* donor cultures (i.e., equivalent or excess recipient cells).
  For A. baumannii and possibly other bacteria, an additional donor expressing RP4 transfer machinery (i.e., pRK2013 or pEVS104) is also required to obtain transconjugants; the reason for this requirement is unclear.
5 Add 700 µl LB (or recipient‐appropriate medium) to a microcentrifuge tube. Add 100 µl of each of the three cultures for a total volume of 1 ml. Centrifuge 2 min at 7000 × *g*, room temperature.
  Ratios of transposon donor, transposase donor, and recipient cells may need to be altered for optimal transposition efficiency. If necessary, the recipient cells can be pelleted and resuspended in less medium to increase the density of cells in 100 µl. In this example, recipient and donor strains are all grow in LB to around the same density.
  Use the optimal recipient growth medium to combine and wash cultures unless this would be harmful to E. coli, in which case a compromise medium that is not harmful to either should be used. In that case, cells can be pelleted by centrifugation first and resuspended in the appropriate medium before proceeding with this step.
  Centrifugation at low speeds results in a looser pellet that may make it less likely that pili required for conjugation are damaged during pipetting. Prewarming the medium to the growth temperature of the recipients may also increase efficiency.
6 Remove supernatant by pipetting and resuspend pellet in 1 ml of LB (or recipient‐appropriate medium). Centrifuge 2 min at 7000 × *g*.
7 Repeat the above wash step.
8 Remove supernatant and resuspend pellet in 30 µl LB (or recipient‐appropriate medium). Pipet the cells onto a cellulose filter placed on a prewarmed LB + 300 µM DAP plate (or recipient‐appropriate/compromise medium). Incubate for ∼2‐24 hr at 37°C (or recipient appropriate temperature).
  Use of the filter is optional but it makes recovery of the cells in the step 9 easier. If no filters are available, spot culture directly on the plate.
  Use a plate with the optimal recipient growth medium to incubate the filters unless this would be harmful to E. coli, in which case a compromise medium that is not harmful to either should be used.
  Incubate at a temperature that is appropriate for both E. coli and the recipient organism.
  Incubation time for the conjugation will need to be optimized for each recipient. Testing and comparing the efficiencies of several incubation times is recommended.
  Use of DAP in the plate is optional here. If DAP is included, the donor E. coli strains will be able to grow. If the recipient strain has a much slower growth rate, it may be advantageous to omit or reduce the DAP to prevent overgrowth of the donors.
9 Using ethanol‐sterilized tweezers, transfer the filter to a microcentrifuge tube containing 200 µl 1× PBS. Vortex ∼15 s to dislodge cells from the filter and resuspend in the buffer.
  If a filter was not used in step 8, recover cells by scraping off the plate with a sterile P1000 pipet tip or sterile wooden stick.
10 Plate cells on agar medium that selects for the Mobile‐CRISPRi transposon‐encoded antibiotic resistance gene and recipient (e.g., LB + kanamycin) without DAP. Initially, plate several volumes to determine optimal amount of your recipient organism to plate to obtain isolated colonies.
  Spreading cells evenly across the plate is important here. Areas of high density may have enough DAP from dead cells to support background growth of DAP‐dependent donor cells.
  For recipients with high efficiency of transposition, dilution in 1× PBS or medium will be necessary to obtain isolated colonies and reduce background of residual DAP‐dependent cells. If colony density is high, restreak colonies for isolation on a new' plate.
11 Stock strains in medium + 15% glycerol and store at −80°C.
12 Once recipient strains have been generated, CRISPRi knockdown can be tested by targeting fluorescent proteins (Support Protocol 1) or by observing reduced plating efficiency upon targeting of essential genes (Support Protocol 2).

## Basic Protocol 4 ICE*Bs1* TRANSFER OF MOBILE‐CRISPRi TO BACILLALES

This protocol describes how to integrate the Mobile‐CRISPRi system into the chromosome of a bacterium of interest using the *B. subtilis* integrative and conjugative element (ICE*Bs1*, or conjugative transposon; Table 3). This protocol has been used with members of the Bacillales Firmicutes (e.g., *Bacillus subtilis*, *Staphylococcus aureus*, *Listeria monocytogenes*, and *Enterococcus faecalis*).

**Table 3:** Strains for Mobile‐CRISPRi (ICE*Bs1* version)

Strain	sJMP no.	Antibiotic res.*a*	Selection*b*	Description/reference
*B. subtilis* with ICE	sJMP251	cm^R^, spc^R^ str^S^	LB with 6 µg/ml chloramphenicol	*B. subtilis* ICE‐containing (cm^R^) donor strain
*B. subtilis* with Mobile‐CRISPRi containing ICE	NA*c*	kan^R^, str^S^	LB with 7.5 µg/ml kanamycin	*B. subtilis* mating strain with Mobile CRISPRi (kan^R^) in ICE element
*B. subtilis* with Mobile‐CRISPRi‐containing ICE	sJMP274	kan^R^, str^S^	LB with 7.5 µg/ml kanamycin	*B. subtilis* mating strain with “test” Mobile CRISPRi (kan^R^), *mRFP*, +sgRNA in ICE element
*B. subtilis* with Mobile‐CRISPRi‐containing ICE	sJMP275	kan^R^, str^S^	LB with 7.5 µg/ml kanamycin	*B. subtilis* mating strain with “test” Mobile CRISPRi (kan^R^), *mRFP*, ‐sgRNA in ICE element
*Bacillus subtilis* CAL89	sJMP210	kan^S^, str^R^	LB with 100 µg/ml streptomycin	Recipient strain (select for ICE(kan) on LB + 7.5 µg/ml kanamycin + 100 µg/ml streptomycin)
*Enterococcus faecalis*	sJMP382	kan^S^, str^R^	BHI with 100 µg/ml streptomycin	Recipient strain (select for ICE(kan) on BHI agar + 100 µg/ml kanamycin + 100 µg/ml streptomycin)
*Listeria monocytogenes*	sJMP17	kan^S^, str^R^	BHI with 100 µg/ml streptomycin	Recipient strain (select for ICE(kan) on BHI agar + 50 µg/ml kanamycin + 100 µg/ml streptomycin)
*Staphylococcus aureus*	sJMP18	kan^S^, str^R^	TSB with 100 µg/ml streptomycin	Recipient strain (select for ICE(kan) on TSB agar + 50 µg/ml kanamycin + 100 µg/ml streptomycin)

a Resistance (s) of recipient *E. coli*. cm^R^, chloramphenicol resistance; kan^R^, kanamycin resistance; spc^R^, spectinomycin resistance; str^R^, streptomycin resistance.

b See Reagents and Solutions for LB, BHI, and TSB medium and stock solution recipes.

c To be created with a Mobile‐CRISPRi plasmid (ICE version) expressing an sgRNA targeting your gene of interest using Basic Protocol 2. The *B. subtilis* strains are being made available through the Bacillus Genetic Stock Center (http://www.bgsc.org/); other strains are available from the ATCC.

### Materials

MC medium (see recipe) with 6 µg/ml chloramphenicol
*B. subtilis* containing a chloramphenicol‐marked ICE*Bs1* element and IPTG‐inducible rapI (sJMP251; Table 3)
BMK (competence) medium (see recipe) + 6 µg/ml chloramphenicol
Plasmid DNA (Mobile‐CRISPRi‐encoding plasmids; ICE version)
LB, BHI, and/or TSB medium (see recipes)
Agar plates with strain‐specific medium (LB, BHI, or TSB) and antibiotic (chloramphenicol, kanamycin, and/or streptomycin; see Table 3 for appropriate concentration)
50% (v/v) glycerol
1 M IPTG
Spizizen's medium and agar plates (see recipe)

125‐ml flask
1‐ml deep 96‐well plates, sterile (e.g., USA Scientific 1896‐1000)
AeraSeal sterile breathable film (Sigma A9224)
Microplate orbital shaker (e.g., GeneMate MP4)
Analytical filter funnels (100 ml) with cellulose nitrate (CN) filters (47 mm, 0.2 µm, sterile; e.g., Nalgene 145‐0020) and vacuum source.

### Create ICE donor strains by integrating Mobile‐CRISPRi (ICEBs1 version) into the Bacillus subtilis chromosomal ICEBs1 by natural competence

1 Inoculate 3 ml MC medium + 6 µg/ml chloramphenicol in a sterile culture tube with a single colony of *B. subtilis* containing a chloramphenicol‐marked ICE*Bs1* element and IPTG‐inducible rapI (sJMP251), and incubate ∼12‐16 hr at 37°C with aeration (shaking at 250 rpm or on a roller drum).
  Chloramphenicol selection retains the ICEBs1 element that could otherwise be lost.
2 Dilute the overnight culture to OD_600_ = 0.1 in 30 ml BMK (competence) medium + 6 µg/ml chloramphenicol in a 125‐ml flask and incubate at 37°C with shaking (250 rpm) until OD_600_ ∼1.5.
  It is very important not to overgrow cultures at this step, asovergrown cells are substantially less competent.
3 Mix 120 μl culture with ≥100 ng (∼1‐5 μl) plasmid DNA in a deep 96‐well plate, cover with breathable film, and incubate 10 min at 37°C without shaking, then 2 hr with shaking (900 rpm) on a microplate shaker.
4 Plate cells on LB agar + 7.5 µg/ml kanamycin and incubate 16‐24 hr at 37°C. Plate several amount to obtain isolated colonies and/or restreak for isolation on a new selection plate.
  Do not select for chloramphenicol resistance at this step; the kanamycin marked Mobile‐CRISPRi cassette has replaced the existing ICEBs1 marker.
5 Prepare stock strains in LB + 7.5 µg/ml kanamycin +15% glycerol at −80°C.
  Do not store B. subtilis plates at 4°C, as B. subtilis loses viability at this temperature.

### ICEBs1 transfer of Mobile‐CRISPRi from B. subtilis donor to recipient strains

6 Inoculate 3 ml LB + 3.75 µg/ml kanamycin with a single colony of *B. subtilis* ICE*Bs1*‐CRISPRi donor strain and 3 ml LB (or strain‐specific rich medium) with a single colony of the recipient strain, and incubate until exponential phase (∼2 hr) at 37°C with aeration (shaking at 250 rpm or rotating on a drum roller).
  Adjust the medium and growth temperature of the recipient strain as necessary in this and subsequent steps.
  Adjust start time of recipient cultures if growth rate is significantly different from that of the donor strain. See Table 3 for strain‐specific media and antibiotic concentrations.
  Use half the regular kanamycin concentration (3.75 µg/ml) for B. subtilis donor strains grown in liquid medium.
7 Dilute the starter cultures to OD_600_ = 0.02 in 5 ml LB + 3.75 µg/ml kanamycin for donors or 15 ml LB (or recipient‐specific rich medium) for recipients, and incubate until OD_600_ ∼0.2 (∼1 hr) at 37°C with aeration.
  Adjust volume of recipient culture depending on number of donor cultures (start ∼15 ml culture for each 4 donors).
8 Add 5 µl 1 M IPTG to 5 ml donor cultures (1 mM IPTG) to induce *rapI* expression and continue to incubate all cultures 1 hr at 37°C with aeration.
  Expression of the ICEBs1 anti‐repressor RapI induces conjugation genes found on the ICE element and promotes excision of ICEBs1 from the chromosome.
9 Adjust OD_600_ of cultures to 0.9. For each mating, add 2.5 ml each of donor and recipient cultures to 5 ml Spizizen's medium in a 50‐ml conical tube and vortex to mix.
  Also set up control matings with no recipient (donor only) or no donor (recipient only).
10 Vacuum cell suspension through an analytical filter funnel to collect the cells on a CN filter, add 5 ml Spizizen's medium, and vacuum again to wash the filter.
11 Using flame‐sterilized forceps, transfer the filter to a Spizizen's medium agar plate and incubate at 37°C for 3 hr.
  Adjust mating time if necessary, depending on recipient.
12 Transfer each filter to a 50‐ml conical tube containing 5 ml Spizizen's medium, and vortex to resuspend cells.
13 Plate on LB + kanamycin (7.5 µg/ml or strain‐specific medium and kanamycin concentration) + streptomycin (100 µg/ml) agar plates to select for transconjugants. Adjust volume plated to obtain isolated colonies and/or restreak for isolation on a new selection plate.
  To determine efficiency, also plate dilutions (∼100 µl of 10^‐4^ and 10^‐5^) on LB + kanamycin (to quantify donor cells) and on LB + streptomycin (to quantify recipient cells).
14 Stock strains in LB + 15% glycerol at −80°C.
15 Once recipient strains have been generated, CRISPRi knockdown can be tested by targeting fluorescent proteins (Support Protocol 1) or by observing reduced plating efficiency upon targeting essential genes (Support Protocol 2).

## Support Protocol 1 QUANTIFICATION OF CRISPRi REPRESSION USING FLUORESCENT REPORTERS

This protocol describes how to quantitatively test the function of the CRISPRi system. A fluorescent protein (e.g., mRFP or sfGFP) expression cassette is incorporated into a Mobile‐CRISPRi construct expressing either an sgRNA targeting the fluorescent protein gene or a nontargeting control. Strains with these Mobile‐CRISPRi constructs are grown with inducer, fluorescence is measured on a fluorometer, and fold repression can be calculated. This protocol is useful when initially testing and optimizing Mobile‐CRISPRi in a new bacterium. (Reference publications for example data for this procedure: Banta et al., 2020; Peters et al., 2019.)

### Materials

LB medium and plates (or medium appropriate for your bacterium)
IPTG (CRISPRi‐Tn*7* version) or anhydrotetracycline (aTc; CRISPRi‐ICE*Bs1* version)
1× PBS (e.g., Fisher BP399500, diluted 1:10)

1‐ml deep 96‐well plates (e.g., USA Scientific 1896‐1000) or culture tubes, sterile
Multichannel pipets (e.g., Rainin Pipet‐lite multi 17013810 and 17013807)
AeraSeal sterile breathable film (Sigma A9224)
Microplate orbital shaker (e.g., GeneMate MP4)
Centrifuge capable of spinning 96‐well plates or culture tubes (e.g., Eppendorf 5920R)
96‐well clear‐bottom black microplates (e.g., Corning C3631)
Microplate fluorometer (e.g., Tecan Infinite 200 PRO Mplex)

### Construct strains with a fluorescent reporter to test CRISPRi knockdown

1 Transfer CRISPRi system with fluorescent reporter (see Table 1) to recipient strain according to Basic Protocol 3 (Tn*7* transfer to Gram‐negative bacteria) or 4 (ICE*Bs1* transfer to Bacillales).
  You will need at least three strains: one encoding the fluorescent protein and an sgRNA targeting the fluorescent protein encoding gene, another encoding the fluorescent protein and a nontargeting sgRNA, and a third, nonfluorescent strain.

### Grow strains to test function of CRISPRi system

2 Add 300 µl LB (or appropriate medium) to wells of a deep 96‐well plate. Inoculate wells with a single colony. Test three or four isolates per strain. Cover plate with sterile breathable film and incubate ∼16 hr at 37°C (or appropriate temperature for the cells) shaking at 900 rpm on a microplate shaker.
  As an alternative, cultures can instead be grown in sterile culture tubes. Adjust medium and growth conditions for your organism if necessary.
3 Serially dilute cultures 1:10,000 into 300 µl medium using a multichannel pipet as follows: dilute cultures 1:100 into LB (or appropriate medium), then dilute 1:100 again into LB (or appropriate medium) containing 0 or 1 mM IPTG (for CRISPRi‐Tn*7* version) or 0 or 0.1 µg/ml aTc (for CRISPRi‐ICE*Bs1* version). Cover plate with sterile breathable film and incubate for ∼10 doublings at 37°C (or appropriate temperature) shaking at 900 rpm on a microplate shaker.
  Additional cultures can be started in extra wells in the plate to monitor cell growth over time by measuring OD_600_. Under these conditions, growth time for E. coli with a ∼40‐min doubling time would be ∼7 hr. Vary medium and incubation temperature and time according to your organism. Cell growth can instead be performed in culture tubes. The concentration of IPTG or aTc can also be varied to determine the induction of the CRISPRi system over a range of inducer concentrations. Substantial culture dilutions are required to accurately quantify the CRISPRi effect due to accumulation of mRFP or sfGFP in overnight cultures.

### Measure fluorescence to determine efficacy of CRISPRi system

4 Spin down cultures in plates for 10 min at 4000 × *g*, room temperature. Decant medium, and resuspend pellet in an equal volume of 1× PBS (∼300 µl) by pipetting up and down ∼10 times.
  Cells can be centrifuged at between 3000 and 4500 × g. Adjust time to ensure pellet formation.
5 Transfer 200 µl cells into a 96‐well clear black‐bottom microplate. In a fluorescence microplate reader, measure cell density (OD_600_) and fluorescence (excitation 485 nm, emission 510 nm for sfGFP; excitation 584 nm, emission 607 nm for mRFP).
6 Analyze knockdown efficacy by first calculating fluorescence/OD_600_ and then calculating a ratio of value of strains with and without the targeting sgRNA.
  Be sure to subtract uninoculated medium background measurement from OD_600_ measurement and nonfluorescent strain background measurement from fluorescent strain measurements.

## Support Protocol 2 TESTING GENE ESSENTIALITY USING CRISPRi SPOT ASSAYS ON PLATES

This protocol describes how to test for gene essentiality using CRISPRi. Serial dilutions of strains with Mobile‐CRISPRi‐encoding sgRNAs targeting genes of interest are spotted on agar plates with various concentrations of inducer. This protocol is useful when initially determining essentiality and for optimizing level of induction of Mobile‐CRISPRi needed for partial knockdown for subsequent experiments.

### Materials

LB medium and agar plates (or medium appropriate for your bacterium), with additions of DAP, IPTG, and/or other additives as appropriate
IPTG (CRISPRi‐Tn*7* version) or anhydrotetracycline (aTc; CRISPRi‐ICE version)

1‐ml deep 96‐well plates (e.g., USA Scientific 1896‐1000) or culture tubes, sterile
AeraSeal sterile breathable film (Sigma A9224)
Sterile 96‐well V‐bottom microplates (e.g., Corning 3896)
Microplate orbital shaker (e.g., GeneMate MP4)
Multichannel pipets (e.g., Rainin Pipet‐lite multi 17013810 and 17013807)
150 × 15‐mm Petri plates (e.g., Falcon 1351058)

1 Construct strains with targeting and nontargeting (control) sgRNAs according to Basic Protocol 2 followed by either Basic Protocol 3 or 4.
2 Add 300 µl LB (or appropriate medium) to wells in a row of a deep 96‐well plate. Inoculate wells with a single colony. Cover plate with sterile breathable film and incubate ∼16 hr at 37°C (or appropriate temperature), shaking at 900 rpm on a microplate shaker.
  As an alternative, cultures can instead be grown in sterile culture tubes. Throughout this protocol, adjust medium, inducer concentration, temperature, and incubation time for your organism if necessary.
  Use of biological replicates (two cultures of the same strain), redundant sgRNAs (multiple strains with different sgRNAs targeting the same gene), and/or technical replicates (spotting the same dish of cultures onto multiple plates twice) is recommended.
3 Using a multichannel pipet, add 90 µl LB medium to all wells of a sterile V‐bottom 96‐well microplate. Add 10 µl of each culture to the wells in row A, mix by pipetting 10 times, and serially dilute (10 µl culture + 90 µl LB medium) into rows B‐H, mixing each time.
4 Prepare 150 × 15‐mm petri plates with LB agar + 0, 10, 100, and 1000 µM IPTG (CRISPRi‐Tn*7* version) or LB + 0, 0.0001, 0.001, and 0.1 µg/ml aTc (CRISPRi‐ICE version). Prewarm plates and ensure that the plate surface is not wet.
5 Using a multichannel pipet, spot 3 µl of each dilution onto the plate. When spot has soaked in, invert plates and incubate ∼16 hr at 37°C.
  To spot the cultures, hold the pipet a few millimeter above the surface of the plate. Depress the plunger to the first stop to expel the drop; it should transfer to the plate (do not push past the first stop or “blow out”; this could contaminate adjacent areas of the plate). Dry the surface of the plate either in a laminar‐flow cabinet or by leaving it slightly cracked in an incubator. Using larger petri plates will allow spotting of an entire 96‐well plate.
6 Image and analyze culture growth on plates. Reduced growth compared to the control (nontargeting sgRNA) will indicate reduced fitness of strains in which CRISPRi targets essential genes. Using plates with a range of IPTG concentrations may aid in selecting an inducer concentration appropriate for partial or complete knockdown.

## Support Protocol 3 TRANSFORMATION OF *E. coli* BY ELECTROPORATION

This protocol details the preparation of electrocompetent *E. coli* cells for transformation as well as the electroporation procedure. While either electrocompetent or chemically competent cells can be used for the protocols detailed here, electroporation is generally of higher efficiency and faster for small‐scale experiments, but requires specialized equipment and is salt sensitive. In this protocol, electroporation is appropriate for transforming ligations into a cloning strain.

### Materials

*E. coli* strains to be transformed
LB medium and agar plates (with selective antibiotics as necessary)
5% and 15% glycerol, autoclaved
SOC medium

2‐L baffled flask, sterile
Spectrophotometer capable of measuring OD_600_ (e.g., Thermo Genesys 30)
Centrifuge able to spin 250‐ml bottles and 1‐ml conical tubes at 4°C (e.g., Eppendorf 5920R)
Disposable cuvettes (e.g., Fisher 14‐955‐127)
250‐ml polypropylene centrifuge bottles (e.g., Nalgene 3120‐0250)
15‐ml conical centrifuge tubes, sterile
1.5‐ml microcentrifuge tubes, sterile
Electroporation cuvettes, 0.1‐cm gap (e.g., Fisher FB101)
Electroporator (e.g., Bio‐Rad Gene Pulser Xcell or Bio‐Rad Micropulser)

### Preparation of electrocompetent E. coli cells

1 Streak strains onto LB agar plates from −80°C stocks to obtain isolated colonies. Incubate ∼14‐18 hr at 37°C.
  Include 300 µM DAP in plates and liquid medium if growing a DAP‐dependent strain.
2 Place 5 ml LB medium in a culture tube, inoculate with a single colony, and incubate ∼14‐18 hr at 37°C with aeration (shaking at 250 rpm or on a drum roller).
3 Place 500 ml LB medium in a 2‐L baffled flask, inoculate with the 5 ml culture (starting OD_600_ ∼0.03), and incubate until mid‐exponential phase (OD_600_ ∼0.3‐0.4, ∼2 hr) at 37°C shaking at 250 rpm.
  Do not overgrow culture. While culture is growing, prechill centrifuge and rotor, and label and prechill centrifuge bottles, tubes, and solutions. Perform remaining steps quickly and keeping cells chilled/on ice.
4 Swirl flask in a pan with an ice/water slurry for ∼5 min to quickly cool the culture.
5 Pellet the cells in 250‐ml polypropylene bottles by centrifuging 10 min at 4000 × *g*, 4°C.
  In this and subsequent steps, cells can be centrifuged between 3000 and 6500 × g depending on centrifuge. Adjust time to minimize centrifugation time but ensure pellet formation.
6 Pour off supernatant and, while holding bottle in ice, resuspend cells in 2 ml 5% glycerol and then add 250 ml 5% glycerol, mix gently by inversion, and pellet the cells by centrifuging 10 min at 4000 × *g*, 4°C.
7 Pour off supernatant, resuspend cells in 2 ml 5% glycerol and then add 125 ml 5% glycerol, mix gently by inversion, and pellet the cells by centrifuging 10 min at 4000 × *g*, 4°C.
8 Pour off supernatant, resuspend cells in 5 ml 15% glycerol, transfer all 10 ml to a single 15‐ml centrifuge tube, and pellet the cells by centrifuging 10 min at 4000 × *g*, 4°C.
9 Pipet off supernatant, resuspend cells in 2.5 ml 15% glycerol, and, on ice, transfer 210‐µl aliquots of cells to each tube (∼13‐14 tubes, about four 50‐µl transformations/tube) and freeze at −80°C.
  Final resuspension volume can be adjusted from ∼1‐5 ml/500 ml starting culture based on the number of cells needed per 50‐µl volume per electroporation.
  Distribute into single‐use aliquots. Do not refreeze unused cells once thawed, but rather adjust aliquot size based on projected needs.

### Transformation of E. coli by electroporation

10 Thaw electrocompetent *E. coli* cells ∼5 min on ice.
11 Prewarm two or three selective plates (i.e., LB agar + appropriate antibiotics and additives) for each transformation to 37°C.
12 Transfers 50‐µl aliquots of cells to 1.5‐ml microcentrifuge tubes on ice.
13 Add up to 1 µl ligation or purified plasmid DNA to cells and transfer to a 0.1‐cm‐gap electroporation cuvette on ice.
  Avoid introducing air bubbles into the suspension, which can result in an electrical discharge that reduces cell viability. It can be effective to set your pipet for several microliters less than the actual volume when transferring. If a bubble may have been introduced, tap cuvette on the counter several times. Ligation reactions contain salt, which may cause arcing if >1 µl of unpurified ligation is electroporated.
14 Set electroporator to exponential decay pulse, 25 µF, 200 Ω, 1.8 kV (i.e., “Bacterial 1” preset on Bio‐Rad Gene Pulser Xcell or “EC1” on Bio‐Rad Micropulser).
  Follow the manufacturer's instructions for electroporation of E. coli for your electroporator.
15 Wipe cuvette dry with a Kimwipe, place in the holder, and then push the pulse button.
16 After completion of pulse, remove cuvette from the holder, add 800 µl SOC medium to the cuvette, mix by pipetting, and transfer cells and medium to a culture tube.
  LB medium can also be used for outgrowth but may reduce transformation efficiency. Prewarming medium to 37°C and/or adding medium to cells as quickly as possible after the pulse may increase efficiency of transformation.
17 Incubate cultures 1 hr at 37°C shaking at 250 rpm or on a roller drum.
18 Plate transformation on two or three prewarmed selective plates to obtain isolated colonies. Incubate plates ∼16 hr at 37°C before proceeding with the rest of your experiment.
  The amount to plate will depend on the competency of the cells. If the transformation efficiency is not known, plate several amounts (e.g., 200, 20, and 2 µl). If isolated colonies are not obtained, restreak for isolation on new plates. Alternatively, plate 80 µl of the transformation and then pellet the remaining cells (∼2 min at ∼6000 × g), spot on a plate, and then streak from that spot to obtain isolated colonies.

## Support Protocol 4 TRANSFORMATION OF CaCl_2_‐COMPETENT *E. coli*

This protocol details the preparation of CaCl_2_‐competent *E. coli* cells for transformation as well as the heat‐shock procedure for transforming these cells. This method requires less specialized equipment than transformation by electroporation and is of lower efficiency but can easily be adapted to be higher throughput when many strains need to be constructed at once, such as when transferring intact plasmids to a mating strain.

### Materials

*E. coli* strains to be transformed
LB medium and agar plates (with selective antibiotics as necessary)
50 mM CaCl_2_ with 10 mM Tris, pH 7.5
50 mM CaCl_2_ with 10 mM Tris, pH 7.5, and 15% glycerol
SOC medium

Spectrophotometer capable of measuring OD_600_ (e.g., Thermo Genesys 30)
Disposable cuvettes (e.g., Fisher 14‐955‐127)
500‐ml baffled flask, sterile
50‐ml conical centrifuge tubes, sterile
Centrifuge able to spin 50‐ml conical tubes at 4°C (e.g., Eppendorf 5920R)
15‐ml conical centrifuge tubes, sterile
Sterile 96‐well V‐bottom microplates (e.g., Corning 3896)
1.5‐ml microcentrifuge tubes, sterile
0.2‐ml PCR strip tubes or 96‐well PCR plates, sterile (for higher throughput transformation)
AeraSeal sterile breathable film (Sigma A9224)
Heat block or thermal cycler (for transformation in strip tubes or 96‐well plates) or water bath (for transformation in microcentrifuge tubes)
Foil seals (e.g., Microseal “F” Foil Bio‐Rad MSF1001)
Multichannel pipets (e.g., Rainin Pipet‐lite multi 17013810 and 17013807)
Microplate orbital shaker (e.g., GeneMate MP4)

### Preparation of chemically competent E. coli cells

1 Streak strains onto LB agar plates from −80°C stocks to obtain isolated colonies. Incubate ∼14‐18 hr at 37°C.
2 Place 5 ml LB medium in a sterile culture tube, inoculate with a single colony, and incubate ∼14‐18 hr at 37°C with aeration (shaking at 250 rpm or on a drum roller).
3 Place 100 ml LB in a sterile 500‐ml baffled flask, inoculate with 1 ml culture (starting OD_600_ ∼0.03), and incubate until mid‐exponential phase (OD_600_ ∼0.3‐0.4, ∼2 hr) at 37°C shaking at 250 rpm.
  Adjust size of culture as needed. 100 ml culture will prepare enough competent cells for two 96‐well plates with 35 µl cells/well.
  Do not overgrow culture. While culture is growing, prechill centrifuge and rotor, and label and prechill centrifuge bottles, tubes, and solutions.
4 Swirl flask in a pan with an ice/water slurry ∼5 min to quickly cool the culture.
5 Pellet the cells in sterile 50‐ml conical centrifuge tubes by centrifuging 10 min at 4000 × *g*, 4°C.
  In this and subsequent steps, cells can be centrifuged between 3000 and 6500 × g depending on the centrifuge. Adjust time to minimize centrifugation time but ensure pellet formation.
6 Pour off supernatant and, while holding tubes in ice, resuspend cells in 25 ml 50 mM CaCl_2_/10 mM Tris, pH 7.5. Mix gently by inversion and place on ice for 15 min.
7 Pellet the cells by centrifuging 10 min at 4000 × *g*, 4°C.
8 Pour off supernatant and, while holding tubes in ice, resuspend cells from each tube in 3.3 ml 50 mM CaCl_2_/10 mM Tris, pH 7.5/15% glycerol, combine resuspended cells into one tube, and place on ice for 30 min.
9 For high‐throughput transformations, transfer 35 µl per well to wells of two sterile 96‐well PCR plates on ice, cover with a foil seal, and freeze at −80°C. Alternatively, transfer 650 µl cells per tube to 1.5‐ml tubes (∼10‐11, tubes each enough for ∼16 transformations [35 µl]) and freeze at −80°C.
  Distribute into single‐use aliquots. Do not refreeze unused cells once thawed, but rather adjust aliquot size based on projected needs.

### Transformation of chemically competent E. coli

10 Thaw CaCl_2_ competent *E. coli* cells on ice ∼5 min and transfer 35‐µl aliquots to sterile 0.2‐ml PCR strip tubes or a 96‐well PCR plate.
  This protocol details performing the transformation in microtiter format to ease handing of a large number of samples, but it can also be performed in individual microcentrifuge and culture tubes.
11 Add 1‐2 µl plasmid DNA (>10 ng) to cells and gently pipet to mix. Close strip caps or cover 96‐well plate with adhesive foil.
12 Hold on ice for 30 min, incubate for exactly 2 min in a 42°C heat block, and then hold on ice for 5 min.
  Alternatively, a thermal cycler can be programmed to cycle through these temperatures, substituting the hold on ice with incubation at 4°C.
13 Add 100 µl SOC medium to a sterile 96‐well microplate. Using a multichannel pipet, transfer cells into plate with SOC and gently pipet to mix. Cover with breathable membrane and incubate 1 hr at 37°C, shaking at 900 rpm on a microplate shaker.
14 Spot transformation onto a prewarmed selective plate and streak out to obtain isolated colonies. Incubate plates ∼16 hr at 37°C before proceeding with the rest of your experiment.
  Alternatively, for high‐throughput transformations, 30 µl of the transformation can be transferred to a 96‐well microplate containing 100 µl SOC medium plus selective antibiotic(s), incubated ∼12‐16 hr at 37°C with shaking at 900 rpm on a microplate shaker, and then pinned onto selective agar plates using a 96‐well pin tool or pinning robot.

## REAGENTS AND SOLUTIONS

Prepare reagents, media, and solutions with purified water (e.g., Milli‐Q). Sterilize all solutions. Some can be autoclaved (as indicated below) for 20‐50 min, liquid cycle. For the others, filter sterilize through a 0.2‐µm‐pore‐size filter: syringe filter (e.g., Fisher 09‐719C), Steriflip (Sigma SCGP00525), or bottle‐top filter (e.g., Nalgene 595‐4520). In general, these media and solutions can be stored indefinitely at room temperature.

### BMK (competence) medium

Combine all components (Table 4) and filter sterilize.

**Table 4:** BMK (Competence) Medium Composition

Name	Formula	MW	Per liter	
Potassium phosphate dibasic	K_2_HPO_4_	174.2	10.7 g	Sigma P8281
Potassium phosphate monobasic	KH_2_PO_4_	136.1	5.2 g	Sigma P0662
d‐(+)‐Glucose	C₆H₁₂O₆	180.2	20 g	Sigma D9434
Trisodium citrate dihydrate	Na_3_C_6_H_5_O_7_·2H_2_O	294.1	0.88 g	Fisher S279‐500
Ferric ammonium citrate	C_6_H_8_FeNO_7_	262.0	0.022 g	Sigma F5879
Potassium aspartate	C_4_H_5_K_2_NO_4_	209.3	2.5 g	Sigma A6558
Magnesium sulfate heptahydrate	MgSO_4_·7H_2_O	246.5	10 ml 1 M stock*a* (10 mM)	Sigma M1880
Manganese chloride	MnCl_2_·4H_2_O	197.9	0.5 ml 300 µM stock*a* (150 nM)	Sigma M3634
l‐Tryptophan	C_11_H_12_N_2_O_2_	204.2	40 mg	Fisher BP395‐100
Yeast extract			0.5 g	BD 212750

a See Reagents and Solutions for stock solution recipes.

### Brain‐heart infusion (BHI) medium (BD 299070)

Dissolve 38 g/L in H_2_O and autoclave. For plates, add 15 g/l agar (e.g., BD 214530) before autoclaving.

### dl‐Dithiothreitol, 100 mM

Dissolve 0.154 g DTT (MW 154.3; Sigma D9779) in 9 ml dH_2_O, adjust volume to 10 ml, filter sterilize, aliquot, and store at −20°C.

### Glucose, 20% (w/v)

Dissolve 200 g glucose **(**Sigma D9434) in 800 ml dH_2_O, adjust volume to 1 liter, and filter sterilize.

### Glycerol, 5% (v/v)

Add 50 ml glycerol (Fisher BP2291) to 950 ml dH_2_O and autoclave.

### Glycerol, 50% (v/v)

Add 250 ml glycerol to 250 ml deionized H_2_O and stir ∼10 min to mix. Aliquot 100 ml/bottle and autoclave.

### Lysogeny broth (LB, Lennox; BD 240230)

Dissolve 20 g/L in H_2_O and autoclave. For plates, add 15 g/l agar (e.g., BD 214530) before autoclaving.

### Magnesium chloride (MgCl_2_), 1 M

Dissolve 20.3 g MgCl_2_·6H_2_O (Sigma M2670) in 80 ml deionized H_2_O, adjust volume to 100 ml, and autoclave or filter sterilize.

### Manganese chloride (MnCl_2_), 300 mM

Dissolve 5.9 g MnCl_2_·2H_2_O (Sigma M3634) in 8 ml dH_2_O, adjust volume to 10 ml. Filter sterilize.

### Manganese chloride (MnCl_2_), 300 µM

Dilute 300 mM MnCl_2_ 1:1000 (10 µl in 10 ml sterile dH_2_O).

### Magnesium sulfate (MgSO_4_), 1 M

Dissolve 24.6 g MgSO4·7H_2_O **(**Sigma M1880) in 80 ml dH_2_O, adjust volume to 100 ml, and autoclave or filter sterilize.

### MC medium

Combine all components (Table 5) and filter sterilize.

**Table 5:** MC Medium Composition

Name	Formula	MW	Per liter	
Potassium phosphate dibasic	K_2_HPO_4_	174.2	10.7 g	Sigma P8281
Potassium phosphate monobasic	KH_2_PO_4_	136.1	5.2 g	Sigma P0662
d‐(+)‐Glucose	C₆H₁₂O₆	180.2	20 g	Sigma D9434
Trisodium citrate dihydrate	Na_3_C_6_H_5_O_7_·2H_2_O	294.1	0.88 g	Fisher S279‐500
Ferric ammonium citrate	C_6_H_8_FeNO_7_	262.0	0.022 g	Sigma F5879
Casamino acids			1.0 g	BD 223050
Potassium glutamate monohydrate	C_5_H_10_KNO_5_·H_2_O	203.2	2.2 g	Sigma G1501
Magnesium sulfate heptahydrate	MgSO_4_·7H_2_O	246.5	20 ml 1 M stock*a* (20 mM)	Sigma M1880
Manganese chloride	MnCl_2_·4H_2_O	197.9	1 ml 300 µM stock*a* (300 nM)	Sigma M3634
l‐Tryptophan	C_11_H_12_N_2_O_2_	204.2	20 mg	Fisher BP395‐100

a See Reagents and Solutions for stock solution recipes.

### Medium additives

Prepare additives (Table 6), filter sterilize each additive, and divide into aliquots before storage. Sterilized medium should be cooled below 55°C before these reagents are added.

**Table 6:** Medium Additives

Reagent	Abbr.	[Stock]	Solvent	Storage	Product no.	Notes
Ampicillin	amp	100 mg/ml	dH_2_O	–20°C	Fisher BP17605	1000× stock for *E. coli*
Anhydrotetracycline	aTc	0.1 mg/ml	DMSO	–20°C	Sigma 37919	use at 0.001‐0.1 µg/ml
Chloramphenicol	cm	20 mg/ml	80% ethanol	–20°C	Sigma C1919	1000× stock for *E. coli*
Diaminopimelic acid	DAP	30 mM (MW 190.2)	dH_2_O	4°C	Sigma 33240	100× stock for *dap^–^ E. coli*
Gentamycin	gent	15 mg/ml	dH_2_O	–20°C	Sigma G1264	1000× stock for *E. coli*
Isopropyl β‐d‐1‐thiogalactopyranoside	IPTG	1 M (MW 238.3)	dH_2_O	–20°C	Sigma I6758	Light sensitive; use at 1‐1000 µM
Kanamycin	kan	30 mg/ml	dH_2_O	–20°C	Fisher BP906	1000× stock for *E. coli*
Spectinomycin	spec	50 mg/ml	dH_2_O	–20°C	Sigma S4014	1000× stock for *E. coli*
Streptomycin	strep	100 mg/ml	dH_2_O	–20°C	Sigma S1277	1000× stock for *E. coli*

### Potassium chloride (KCl), 1 M

Dissolve 7.5 g KCl (Sigma P9333) in 80 ml deionized H_2_O, adjust volume to 100 ml, and autoclave or filter sterilize.

### SOC medium

Dissolve 4 g tryptone (BD 211705), 1 g yeast extract (BD212750), 0.1 g NaCl (Fisher S271), and 500 µl 1 M KCl (see recipe) in 200 ml H_2_O in a bottle with a stir bar. Autoclave to sterilize. When solution has cooled to below ∼60°C, add 1 ml sterile 1 M MgCl_2_ (see recipe) and 3.6 ml sterile 20% (w/v) glucose (see recipe) and stir to mix. Aliquot 10 ml/tube and store between −20°C and 25°C.

### Spizizen's medium and agar plates

Combine all components listed in Table 7 and filter sterilize.

**Table 7:** Spizizen's Medium Composition*a*

Name	Formula	MW	Per liter	
Potassium phosphate dibasic	K_2_HPO_4_	174.2	14 g	Sigma P8281
Potassium phosphate monobasic	KH_2_PO_4_	136.1	6 g	Sigma P0662
Ammonium sulfate	(NH_4_)_2_SO_4_	132.1	2 g	Sigma A4418
d‐(+)‐Glucose	C₆H₁₂O₆	180.2	5 g	Sigma D9434
Trisodium citrate dihydrate	Na_3_C_6_H_5_O_7_·2H_2_O	294.1	1 g	Fisher S279‐500
Magnesium sulfate heptahydrate	MgSO_4_·7H_2_O	246.5	0.2 g	Sigma M1880
l‐Tryptophan	C_11_H_12_N_2_O_2_	204.2	50 mg	Fisher BP395‐100

a For plates, mix 250 ml filter‐sterile 2× medium with 250 ml autoclaved H_2_O + 7.5 g agar, cooled to 55°C, swirl to mix, and pour into 15 × 100‐mm petri dishes (e.g., Corning 351029).

### Tryptic Soy broth (TSB)

Dissolve 30 g/L TSB (BD 211825) in H_2_O and autoclave. For plates, add 15 g/liter agar (e.g., BD 214530) before autoclaving.

## COMMENTARY

### Background Information

CRISPR interference (CRISPRi) is a programmable gene‐knockdown tool based on the clustered regularly interspaced short palindromic repeats (CRISPR) adaptive immune systems found in bacteria and archaea that restrict viral and plasmid DNA and RNA. Several types of CRISPR systems have been co‐opted for use in CRISPRi (Qi et al., 2013; Specht, Xu, & Lambert, 2020; Zheng et al., 2019), but here we will focus on the commonly used Type II‐A system from *Streptococcus pyogenes* (reviewed in Wright, Nuñez, & Doudna, 2016). In the native *S. pyogenes* CRISPR system, a CRISPR array containing spacers that designate target genes is transcribed into a pre‐crRNA (CRISPR RNA), which is subsequently processed into individual crRNAs containing only one spacer. Processed crRNAs form a complex with a tracrRNA (trans‐activating CRISPR RNA) and the CRISPR‐associated nuclease Cas9; this complex is directed to target DNA by base‐pairing between the crRNA spacer and a complementary DNA sequence in the target known as a protospacer. In addition to spacer‐protospacer complementarity, Cas9 requires a short protospacer‐adjacent motif (PAM; NGG for *S. pyogenes*) for DNA binding that prevents self‐targeting of CRISPR arrays that lack this motif. The Cas9‐tracr/crRNA complex binds to the PAM sequence, unzips the DNA duplex, anneals the crRNA and protospacer DNA, and then—if the spacer and protospacer match sufficiently—cleaves both strands of the DNA (Sternberg, Redding, Jinek, Greene, & Doudna, 2014). Doudna, Carpentier, and colleagues first showed that Cas9 is an RNA‐guided endonuclease and simplified the natural dual RNA system by engineering a fused tracr/crRNA known as a single guide RNA (sgRNA; Jinek et al., 2012). Cas9 can thus be programmed to target new genes of interest simply by changing the 20‐nt sgRNA spacer to match a protospacer with an adjacent PAM in the target DNA.

To repurpose CRISPR as a gene‐knockdown technology, Qi and colleagues mutated the two nuclease active sites in Cas9, producing an inactive variant known as dCas9 (“dead Cas9”; Qi et al., 2013). dCas9 retains the ability to be directed to target genes via programmable sgRNAs but can no longer cut DNA. Instead, dCas9 inhibits transcription at the step of initiation or elongation by acting as a steric block to RNA polymerase in bacterial systems. The modest sequence requirements for CRISPRi repression—i.e., an NGG PAM sequence and adjacent spacer (Jinek et al., 2012)—and engineered Cas9 variants with altered‐ or relaxed‐specificity PAM dependencies (Walton, Christie, Whittaker, & Kleinstiver, 2020) suggest that nearly all bacterial genes can be targeted by CRISPRi. CRISPRi systems have been established in many diverse bacteria and have primarily been used to phenotype individual essential genes in proof‐of‐principle work. However, CRISPRi has been increasingly valuable in targeting larger, defined sets of genes (e.g., essential genes; Liu et al., 2017; Peters et al., 2016) and in pooled phenotyping approaches at the genome scale for both model (Rousset et al., 2018; Wang et al., 2018) and nonmodel bacteria (Lee et al., 2019; Yao et al., 2020; reviewed in Vigouroux & Bikard, 2020).

### Critical Parameters and Troubleshooting

#### Optimizing Mobile‐CRISPRi transfer and integration

Distinguishing between Mobile‐CRISPRi transfer and integration problems presents a challenge because both processes are required to obtain transconjugants. The efficiency of Mobile‐CRISPRi transfer and integration varies by strain, such that two strains of the same species can produce vastly different numbers of transconjugants (e.g., *P. aeruginosa* PAO1 and PA14 differ by >100‐fold; Peters et al., 2019). Cell surface features (e.g., capsules) as well as defense systems that destroy horizontally transferred DNA (e.g., restriction enzymes and other CRISPR systems) can reduce transfer and integration efficiency (Thomas & Nielsen, 2005). Extending the mating time to 24 hr may improve recovery of transconjugants for low‐efficiency recipients. Conjugation efficiencies may increase for some recipients after growth at elevated temperatures or heat shock, possibly owing to inactivation of restriction enzymes or other protein‐based inhibitors (Irani & Rowe, 1997; Zeng, Ardeshna, & Lin, 2015).

Transfer, integration, or both can be limiting for obtaining transconjugants from Tn*7*‐ and ICE*Bs1*‐based Mobile‐CRISPRi systems. For instance, Tn*7* systems fail to produce transconjugants when *B. subtilis* 168 is the recipient strain; however, the *B. subtilis att*_Tn_ *_7_* site is functional for integration when cloned into *E. coli*, suggesting that transfer of the two‐plasmid Tn*7* system is limiting in this case (Peters et al., 2019). In contrast, Tn*7* integration into wild‐type *Agrobacterium tumefaciens* occurs at low frequency because insertion into the native *att*_Tn_ *_7_* site disrupts a gene immediately downstream of *glmS*; introducing a second *att*_Tn_ *_7_* site at a neutral locus alleviated this issue (Figueroa‐Cuilan, Daniel, Howell, Sulaiman, & Brown, 2016). If mating fails for Tn*7* Mobile‐CRISPRi, we recommend adding a second copy of the *att*_Tn_ *_7_* site on a plasmid to test for integration into that site either in *E. coli* or in the relevant recipient. Further, we have observed that inclusion of a third donor strain containing a self‐transferrable RP4 helper plasmid—either pRK2013 (Ditta, Stanfield, Corbin, & Helinski, 1980) or pEVS104 (Stabb & Ruby, 2002)—is required to obtain transconjugants when *A. baumannii* is the recipient (Peters et al., 2019); although the mechanism is unknown, inclusion of the RP4 helper may also increase recovery of transconjugants for other recipients as well. We strongly prefer use of pEVS104 as the RP4 helper plasmid due to its *pir*‐dependent origin that does not replicate in recipient strains (pRK2013 has a ColE1 origin which replicates in species related to *E. coli*). For strains in which restriction endonucleases are a major barrier to conjugation, such as *Z. mobilis*, we have used mutants deleted for genes encoding restriction systems to increase recovery of Tn*7* Mobile‐CRISPRi transconjugants by several orders of magnitude (Banta et al., 2020). Tn*7* systems can also be electroporated into cells if mating is inefficient or inconvenient (Choi & Schweizer, 2006). Although the Tn*7* recognition site in the 3′ end of *glmS* is generally conserved across bacteria, the ICE*Bs1* recognition site in the *leu2* tRNA is present only in bacteria related to *B. subtilis* (e.g., various *Bacillus* species, *L. monocytogenes*, and *S. aureus*). However, work from the Voigt lab has shown that a smaller, engineered ICE element (mini‐ICE) can insert at noncanonical sites (Brophy et al., 2018), raising the possibility that the ICE*Bs1* host range is broader than previously appreciated. Therefore, we recommend testing ICE*Bs1* Mobile‐CRISPRi in Gram‐positives that lack an obvious *leu2* gene and mapping the insertion sites using arbitrary PCR (Saavedra, Schwartzman, & Gilmore, 2017).

#### Cloning into Mobile‐CRISPRi modules

Mobile‐CRISPRi vectors are composed of functional “modules” flanked by restriction sites to enable swapping or cloning of new components by restriction digestion and Gibson assembly (Gibson et al., 2009) or ligation. The modules are as follows: (1) antibiotic‐resistance genes (e.g., conferring resistance to kanamycin, chloramphenicol, gentamicin, and spectinomycin) flanked by XhoI sites; (2) reporter genes (e.g., *mRFP*, *sfGFP*) flanked by PmeI sites; (3) an *sgRNA* gene with an existing spacer or BsaI sites to clone new spacers (see Cloning new sgRNA spacers into Mobile‐CRISPRi, above) flanked by EcoRI sites; (4) promoter‐regulation genes (e.g., *lacI* and *tetR*) flanked by SmaI sites; (5) the *dcas9* promoter and ribosome‐binding site (RBS) flanked by SpeI sites, and (6) *dcas9* flanked by SpeI and AscI sites (cutting with SpeI also removes the *dcas9* promoter and RBS module).

#### Optimizing Mobile‐CRISPRi knockdown

CRISPRi knockdown from published Mobile‐CRISPRi vectors varies by strain and is not currently predictable (Peters et al., 2019). If knockdown is insufficient for desired experiments, we recommend testing whether *sgRNA* or *dcas9* expression is limiting by cloning these genes individually onto multicopy plasmids and testing knockdown alongside Mobile‐CRISPRi integrated into the chromosome. We used this strategy to determine that sgRNA expression was limiting during optimization of Mobile‐CRISPRi for *Z. mobilis* (Banta et al., 2020). Optimizing Mobile‐CRISPRi knockdown involves cloning new components into the function modules listed above (specifically, the *sgRNA* and *dcas9* modules 3, 5, and 6). If expression of the sgRNA is limiting, we clone synthetic DNA with a strong promoter and sgRNA gene into the EcoRI sites. If a stronger promoter has not been characterized for the relevant recipient bacterium, we empirically determine the activity of strong promoters from model bacteria (i.e., *E. coli* and *B. subtilis*) in the context of CRISPRi in the recipient. If *dcas9* expression is limiting, we clone synthetic DNA containing a strong promoter and an RBS optimized for the relevant recipient strain into the SpeI sites. We use the RBS Calculator (https://salislab.net/software/) from the Salis lab to estimate RBS efficacy and design sites with higher translation rates (Espah Borujeni et al., 2017). Alternatively, codon‐optimized variants of *dcas9* (along with a new promoter and RBS) can be cloned into the SpeI‐AscI sites of Mobile‐CRISPRi for bacteria with high or low GC content. Overexpression of dCas9 is toxic in some bacteria (Cho et al., 2018; Lee, Hoynes‐O'Connor, Leong, & Moon, 2016; Qu et al., 2019; Rock et al., 2017), even when expressed in single copy from the chromosome (e.g., in *P. aeruginosa*). In these cases, *dcas9* expression must be reduced to avoid pleiotropic effects; we previously used low‐level constitutive promoters from the Anderson promoter series (Qu et al., 2019) to drive *dcas9* expression in *P. aeruginosa*, avoiding toxic effects while maintaining knockdown. The Bikard lab has developed a screening strategy to avoid toxicity from certain sgRNA seed sequences (known as “bad seeds”) by randomizing bases within the *dcas9* RBS to identify constructs that minimize toxicity but still offer robust knockdown (Depardieu & Bikard, 2020).

### Understanding Results

#### Basic Protocol 3: Tn7 Transfer of Mobile‐CRISPRi to Gram‐negative bacteria

Tn*7* Mobile‐CRISPRi transfer and integration efficiency varies by strain. In our experience, efficiency ranges from ∼1% (*E. coli*) to ∼10^–7^% (*P. mirabilis*) with a median efficiency of ∼10^–2^‐10^–3^% (Peters et al., 2019).

#### Basic Protocol 4: ICEBs1 transfer of Mobile‐CRISPRi to Bacillales

ICE*Bs1* Mobile‐CRISPRi transfer and integration efficiency varies by strain. In our experience, efficiency ranges from ∼1% (*B. subtilis*) to ∼10^‐7^% (*E. faecalis*) with a median efficiency of ∼10^‐3^%‐10^‐4^% (Peters et al., 2019).

#### Support Protocol 1: Quantifying CRISPRi repression using fluorescent reporters

Mobile‐CRISPRi knockdown efficacy varies by strain and ranges from 150‐fold knockdown (*S. aureus*) to 8‐fold knockdown (*P. aeruginosa*) with a median efficacy of ∼40‐fold (Peters et al., 2019).

### Time Considerations

The mating process can be performed in a single day, but many other aspects of the protocols listed here are dependent on the growth rate of the recipient strain. For instance, transconjugants can be evaluated the next day when using *E. cloacae* as a recipient, but *Z. mobilis* transconjugants take 3‐4 days to form robust colonies.

### Author Contributions

**Amy B. Banta**: Conceptualization; methodology; writing‐original draft; writing‐review & editing. **Ryan D. Ward**: Software; writing‐original draft; writing‐review & editing. **Jennifer S. Tran**: Visualization; writing‐original draft; writing‐review & editing. **Emily E. Bacon**: Writing‐original draft; writing‐review & editing. **Jason M. Peters**: Conceptualization; funding acquisition; methodology; writing‐original draft; writing‐review & editing.
